# Lymphoma or Hematoma: Emergency Department Recognition of a Diagnostic Pitfall

**DOI:** 10.7759/cureus.107745

**Published:** 2026-04-26

**Authors:** Cayla Guerra, Sierra Peace, Fiona E Gallahue

**Affiliations:** 1 Emergency Medicine, Madigan Army Medical Center, Joint Base Lewis-McChord, USA; 2 Emergency Medicine, University of Washington Medical Center, Seattle, USA

**Keywords:** adult neck mass, cancer emergency medicine, core needle biopsy (cnb), emergency diagnostics, large b cell lymphomas

## Abstract

Neck swelling with upper-extremity edema in a cancer patient presenting to the emergency department (ED) is a concerning clinical picture that may indicate underlying venous obstruction or malignancy. A 74-year-old woman developed a rapidly enlarging left supraclavicular mass after core needle biopsy of a lesion later diagnosed as high-grade follicular large B-cell lymphoma. Follow-up imaging initially suggested a postprocedural hematoma; however, bedside Doppler ultrasound in the emergency department demonstrated internal vascular flow, prompting repeat contrast-enhanced computed tomography (CT), which confirmed aggressive tumor progression with venous compression rather than bleeding. The patient was admitted for emergent inpatient Rituximab, Cyclophosphamide, Doxorubicin, Vincristine, and Prednisone (R-CHOP) chemotherapy, which resulted in rapid symptomatic improvement and complete metabolic remission. This case highlights the diagnostic ambiguity of rapid postbiopsy enlargement of malignant neck masses and underscores the importance of bedside ultrasound, multidisciplinary evaluation, and reconsideration of initial imaging interpretations to avoid delays in definitive therapy.

## Introduction

Neck swelling accompanied by upper-extremity edema is a clinically significant presentation in the emergency department (ED), often signaling underlying vascular obstruction or malignancy [1-4]. In adults, supraclavicular neck masses carry a high likelihood of cancer, and early recognition can markedly influence outcomes [4]. Venous obstruction or occlusion involving the cervical or mediastinal vessels, most commonly the subclavian or internal jugular veins, may result from thrombosis or direct tumor compression and typically presents with arm swelling, pain, or erythema [1-3, 5]. Current guidelines recommend fine-needle aspiration as the first-line diagnostic procedure for neck masses because of its high diagnostic accuracy and extremely low risk of tumor seeding, whereas core or open biopsy is reserved for nondiagnostic cases or when a larger tissue sample is required [6, 7]. Although tumor seeding after biopsy has been described, it remains exceedingly rare, particularly in lymphoid malignancies [6, 8-10]. Nevertheless, recognizing this potential complication is important when evaluating post-biopsy changes in mass size or morphology [8, 9].

Rapid enlargement of a neck mass following biopsy presents both diagnostic and management challenges. The differential diagnosis includes postprocedural hematoma, infection, aggressive tumor proliferation, and, rarely, biopsy-tract seeding [6, 11, 12]. Misattributing true tumor progression to a hematoma may delay definitive therapy, especially when early imaging findings are equivocal. In this case, a patient’s enlarging cervical mass was initially presumed to represent post-biopsy hemorrhage. On re-presentation to the ED with new ipsilateral arm swelling, updated imaging instead revealed rapid tumor progression with venous compression. This report underscores the need for prompt reassessment of presumed postprocedural complications and highlights the diagnostic ambiguity that can accompany rapid postbiopsy enlargement of malignant neck masses [1, 11, 12].

## Case presentation

A 74-year-old woman with a remote history of tobacco use presented to her primary care provider in mid-September 2024 with a new, mildly tender left supraclavicular mass that she had first noticed the day before. This visit occurred approximately two weeks prior to her subsequent ED presentation. She denied fever, chills, night sweats, weight loss, or fatigue. Physical examination revealed a 3-cm, slightly tender mass in the left supraclavicular fossa without other cervical lymphadenopathy.

**Figure 1:**
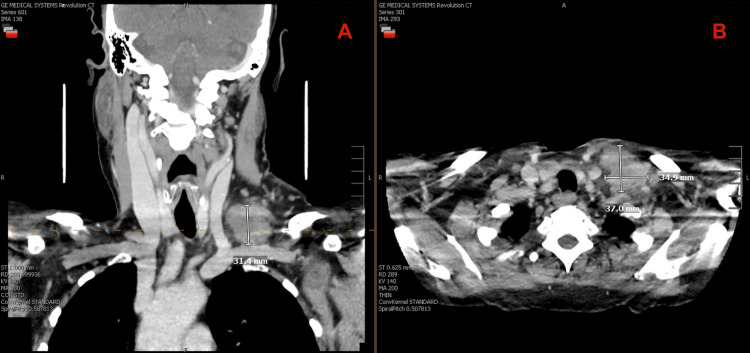
Contrast-enhanced CT of the neck obtained September 12, 2024, prior to biopsy. Coronal (A) and axial (B) views show a left supraclavicular mass measuring approximately 37 × 35 mm, displacing adjacent vascular structures without compression or hemorrhage.

Contrast-enhanced computed tomography (CT) of the neck performed in mid-September demonstrated a 3.5 × 3.7 × 3.1 cm mass in the left supraclavicular region with several enlarged adjacent lymph nodes (Figure 1). CT of the chest, abdomen, and pelvis showed small pulmonary nodules and mild retroperitoneal and mesenteric lymphadenopathy but no definite primary malignancy. Peripheral blood flow cytometry revealed a small atypical B-cell population. Over the next two weeks, the mass remained stable in size. 

An ultrasound-guided core needle biopsy of the mass was then performed, yielding four tissue samples without immediate complication. Pathology confirmed high-grade follicular large B-cell lymphoma with a high proliferation index (Ki-67 70-80%) and germinal-center immunophenotype, according to the oncology notes (no images were provided). Immediate postbiopsy ultrasound demonstrated no evidence of hemorrhage; however, this ultrasound was performed at the bedside and was not saved in the patient’s chart.

**Figure 2:**
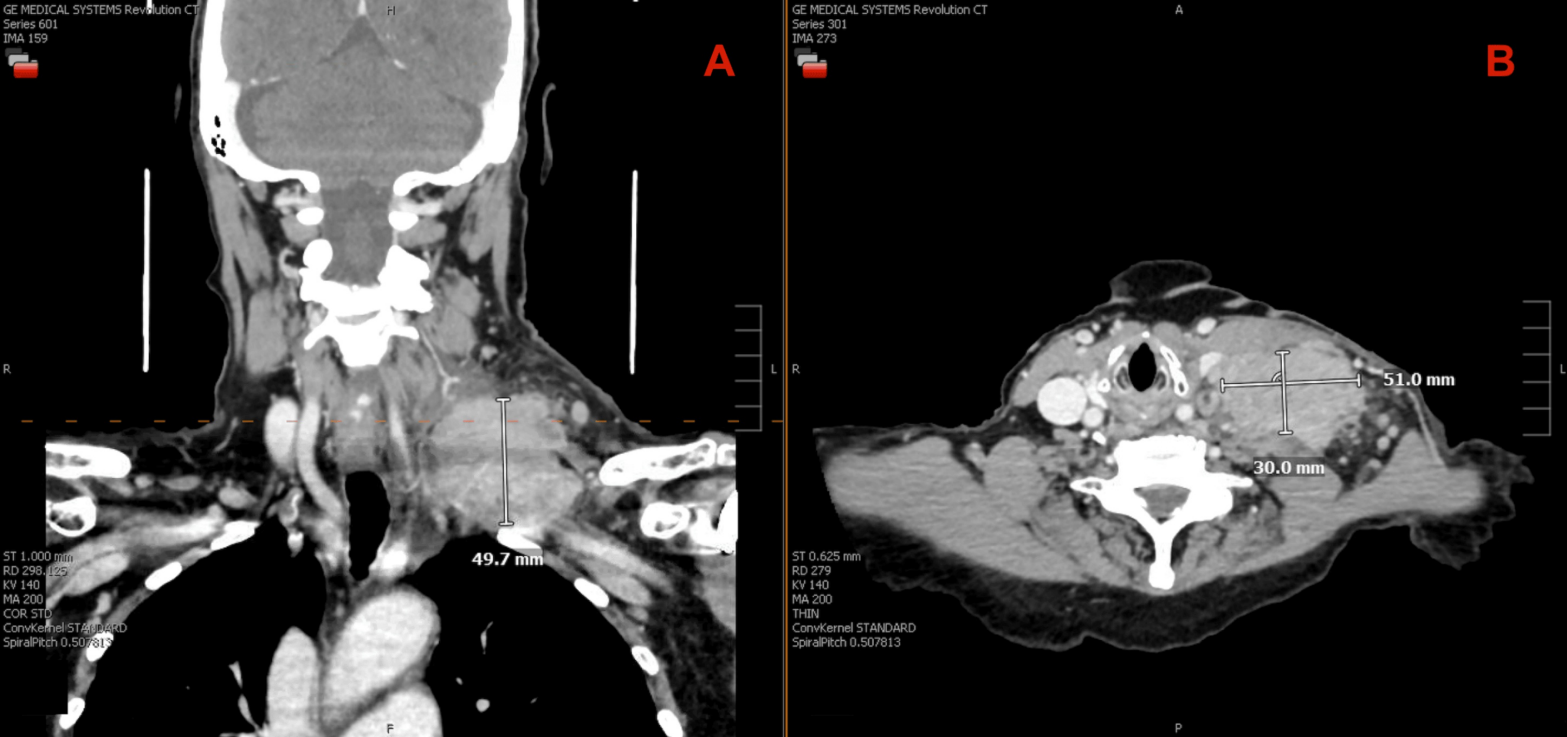
Contrast-enhanced CT of the neck obtained September 30, 2024, approximately two weeks after ultrasound-guided core needle biopsy. Coronal (A) and axial (B) views demonstrate enlargement of the same mass to 49 × 30 mm with mild displacement of adjacent vasculature, presumed to represent a hematoma.

Throughout the two weeks following biopsy, the patient noted rapid enlargement of the neck mass with new left arm swelling and discomfort. The mass was hard, immobile, and only mildly tender to palpation. Outpatient repeat CT in late September demonstrated interval enlargement of the mass to 5.1 × 3.0 × 4.9 cm with mild flattening of the left internal jugular vein (Figure 2). 

The radiologist interpreted this as "probable hematoma in the setting of recent biopsy," and conservative management was recommended by the outpatient oncologist based on this scan.

**Figure 3:**
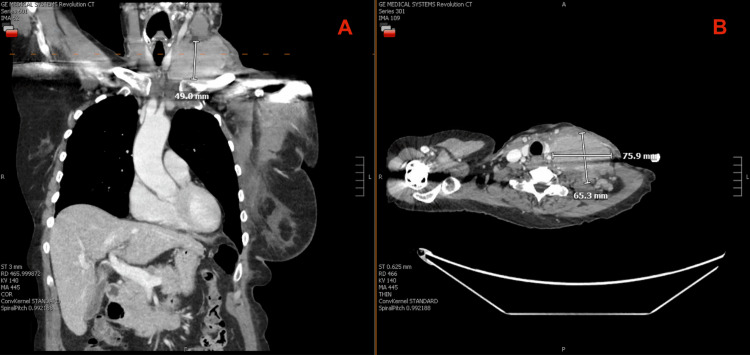
Contrast-enhanced CT of the chest obtained in the ED on October 17, 2024. Coronal (A) and axial (B) CT images demonstrate rapid interval enlargement of the left supraclavicular mass to approximately 76 × 65 mm with vascularity and compression and occlusion of the left internal jugular and subclavian veins, consistent with venous outflow obstruction due to extrinsic tumor compression.

Progressive swelling and neck pressure prompted emergency evaluation in mid-October. In the ED, bedside color Doppler ultrasound demonstrated internal vascular flow within the mass, suggesting a viable tumor rather than a hematoma. Because of this finding, CT imaging with intravenous contrast was obtained, which showed that the mass was well vascularized throughout and had enlarged to approximately 6.5 × 7.6 × 4.9 cm, with displacement and compression of the left internal jugular and subclavian veins, but without intraluminal thrombus or evidence of hemorrhage (Figure 3). These findings established that the enlargement represented aggressive tumor progression with venous compression rather than postprocedural bleeding. 

Given the rapid expansion, the patient was admitted for urgent oncologic management rather than awaiting planned outpatient induction. After discussion with the inpatient oncology team, R-CHOP (Rituximab, Cyclophosphamide, Doxorubicin, Vincristine, and Prednisone) chemotherapy was initiated, leading to marked improvement in swelling and neck pressure within 24 hours. She completed six cycles without major adverse effects. End-of-treatment positron emission tomography/computed tomography demonstrated a Deauville 1 complete metabolic response. At six-month follow-up, she remained in remission with no recurrent symptoms.

## Discussion

Rapid enlargement of a neck mass following biopsy presents a diagnostic dilemma for emergency physicians. Distinguishing postprocedural hematoma from tumor progression or, rarely, tumor seeding can be difficult based on imaging alone [1, 4, 12]. CT may demonstrate an interval increase in size but cannot reliably differentiate hemorrhage from viable tumor, particularly when enhancement and blood products coexist [11]. Consequently, radiologic interpretation often favors hematoma in the setting of recent biopsy [12]. In this case, the mid-September biopsy was followed by late-September CT showing mild flattening of the left internal jugular vein and interval enlargement interpreted as probable hematoma. By mid-October, however, CT venography confirmed marked expansion with venous compression and no evidence of hemorrhage, establishing progressive tumor growth rather than postbiopsy bleeding. The rapid resolution of swelling and venous obstruction within 24 hours of R-CHOP initiation further supported that the enlargement reflected aggressive, chemotherapy-sensitive tumor biology rather than a procedural complication. This is consistent with the patient’s diagnosis of a high-grade follicular lymphoma.

Several reports describe malignant tumors masquerading as hematomas, leading to diagnostic delay and morbidity [11, 12]. Misclassification of rapid tumor progression as a postprocedural change is well recognized in head and neck malignancies, where even brief delays in therapy can affect outcomes [1]. In the present case, immediate postbiopsy ultrasound demonstrated no hemorrhage, an important clue that made a subsequent "hematoma" diagnosis less likely to our team. The turning point occurred in the emergency department, where a point-of-care color Doppler ultrasound revealed internal vascular flow, raising suspicion and prompting CT venography. This step confirmed patent but compressed cervical veins and excluded intraluminal thrombus, findings that redirected the diagnosis toward tumor progression. The case underscores the diagnostic value of bedside ultrasound for emergency physicians evaluating enlarging postbiopsy neck masses.

Rapid postbiopsy enlargement of lymphoma has been described in isolated case reports and small series, most often involving aggressive B-cell subtypes [6, 8-10]. The phenomenon is thought to reflect intrinsic tumor biology, potentially accentuated by local inflammation or altered vascular permeability after biopsy, rather than true procedural seeding [6, 10]. In this patient, the absence of postprocedural bleeding and the gradual, progressive enlargement over several weeks favor biologic proliferation as the cause. The high Ki-67 index (70-80%), consistent with aggressive B-cell lymphoma behavior, further supports this interpretation [10].

True tumor seeding after fine-needle or core biopsy remains extraordinarily rare. When reported, it typically involves solid organ carcinomas or sarcomas, not lymphoid malignancies [6, 10]. The literature and clinical context therefore suggest that this patient’s course represented natural disease progression rather than iatrogenic dissemination. Nonetheless, emergency physicians should remain aware of this theoretical risk and recognize that rapid mass expansion, especially postbiopsy, even after a diagnosis of hematoma, may warrant reconsideration of initial imaging interpretations.

This case offers several lessons relevant to emergency care. First, interval enlargement of a postbiopsy mass should not be automatically attributed to hemorrhage, particularly when prior imaging showed no bleeding [1, 4, 11]. Second, bedside duplex ultrasound provides immediate, actionable data about internal vascularity, guiding the decision to pursue CT venography or early oncologic consultation. Third, the clinical presentation itself was not entirely consistent with a hematoma but rather with an expanding mass. The patient had a firm, immobile lesion that was progressively enlarging without bruising or distal edema prior to vascular compression. These clinical clues may help the ED provider broaden the differential diagnosis despite a presumed diagnosis prior to ED evaluation. Finally, multidisciplinary coordination among emergency medicine, radiology, and oncology is essential for timely diagnosis and treatment. The patient’s rapid improvement after initiation of R-CHOP therapy highlights the impact of prompt recognition and definitive management in aggressive lymphoma.

## Conclusions

Rapid enlargement of a neck mass after biopsy should raise concern for aggressive tumor progression even after a diagnosis of presumed postprocedural hemorrhage, particularly when prior imaging shows no evidence of bleeding. This case demonstrates the dramatic impact of early recognition on outcomes. For emergency physicians, bedside ultrasound provides immediate insight into vascularity and tissue perfusion, guiding advanced imaging and earlier oncologic consultation. Maintaining diagnostic vigilance and initiating multidisciplinary evaluation are essential, as prompt therapy can result in rapid and complete resolution, even in cases of dramatic postbiopsy expansion, as in the case presented here.

## References

[1] Pynnonen MA, Gillespie MB, Roman B (2017). Clinical practice guideline: evaluation of the neck mass in adults. Otolaryngol Head Neck Surg.

[2] Unsal EE, Karaca C, Ensarí S (2003). Spontaneous internal jugular vein thrombosis associated with distant malignancies. Eur Arch Otorhinolaryngol.

[3] Haen P, Mege D, Crescence L, Dignat-George F, Dubois C, Panicot-Dubois L (2019). Thrombosis risk associated with head and neck cancer: a review. Int J Mol Sci.

[4] Ellison E, LaPuerta P, Martin SE (1999). Supraclavicular masses: results of a series of 309 cases biopsied by fine needle aspiration. Head Neck.

[5] Colevas AD, Cmelak AJ, Pfister DG (2025). NCCN Guidelines® insights: head and neck cancers, version 2.2025. J Natl Compr Canc Netw.

[6] Robertson EG, Baxter G (2011). Tumour seeding following percutaneous needle biopsy: the real story!. Clin Radiol.

[7] Nyquist GG, Tom WD, Mui S (2008). Automatic core needle biopsy: a diagnostic option for head and neck masses. Arch Otolaryngol Head Neck Surg.

[8] Kipnis P, Ramanathan D, Hoehn R (2024). Tumor seeding across specialties: a systematic review. Front Oncol.

[9] Berger-Richardson D, Swallow CJ (2017). Needle tract seeding after percutaneous biopsy of sarcoma: risk/benefit considerations. Cancer.

[10] Shah KS, Ethunandan M (2016). Tumour seeding after fine-needle aspiration and core biopsy of the head and neck--a systematic review. Br J Oral Maxillofac Surg.

[11] Lin HJ, Hsu CY, Tsai SC (2021). Cervical malignant teratoma masquerading as a hematoma: a case report. J Int Med Res.

[12] Ward WG Sr, Rougraff B, Quinn R, Damron T, O'Connor MI, Turcotte RE, Cline M (2007). Tumors masquerading as hematomas. Clin Orthop Relat Res.

